# Novel Coronavirus Outbreak in Wuhan, China, 2020: Intense Surveillance Is Vital for Preventing Sustained Transmission in New Locations

**DOI:** 10.3390/jcm9020498

**Published:** 2020-02-11

**Authors:** Robin N. Thompson

**Affiliations:** 1 Mathematical Institute, University of Oxford, Woodstock Road, Oxford OX2 6GG, UK; robin.thompson@chch.ox.ac.uk; 2 Christ Church, University of Oxford, St Aldates, Oxford OX1 1DP, UK

**Keywords:** 2019-nCoV, mathematical modelling, infectious disease epidemiology, major outbreak, forecasting, coronavirus, Wuhan, SARS

## Abstract

The outbreak of pneumonia originating in Wuhan, China, has generated 24,500 confirmed cases, including 492 deaths, as of 5 February 2020. The virus (2019-nCoV) has spread elsewhere in China and to 24 countries, including South Korea, Thailand, Japan and USA. Fortunately, there has only been limited human-to-human transmission outside of China. Here, we assess the risk of sustained transmission whenever the coronavirus arrives in other countries. Data describing the times from symptom onset to hospitalisation for 47 patients infected early in the current outbreak are used to generate an estimate for the probability that an imported case is followed by sustained human-to-human transmission. Under the assumptions that the imported case is representative of the patients in China, and that the 2019-nCoV is similarly transmissible to the SARS coronavirus, the probability that an imported case is followed by sustained human-to-human transmission is 0.41 (credible interval [0.27, 0.55]). However, if the mean time from symptom onset to hospitalisation can be halved by intense surveillance, then the probability that an imported case leads to sustained transmission is only 0.012 (credible interval [0, 0.099]). This emphasises the importance of current surveillance efforts in countries around the world, to ensure that the ongoing outbreak will not become a global pandemic.

## 1. Introduction

The infectious agent driving the ongoing pneumonia outbreak (the 2019-nCoV) appears to have transitioned from animals into humans, with the Huanan seafood wholesale market in Wuhan, China, representing the most likely source [1,2,3,4,5]. Since then, cases have been recorded in other countries, and initial estimates suggest a hospital fatality risk of around 14% [6], although estimates of disease severity early in an outbreak are often imprecise [7,8,9]. Even countries without confirmed cases have been on high alert. For example, even prior to the two cases in the United Kingdom on 31 January 2020, officials were reported to be attempting to trace as many as 2000 visitors that had travelled to that country from Wuhan [10].

The most devastating infectious disease outbreaks are those that have a wide geographical range, as opposed to being confined to a small region [11,12]. The previously known virus that is most similar to the 2019-nCoV is the SARS coronavirus [13], which generated cases in 37 countries in 2002–2003 [13,14]. Since the 2019-nCoV is clearly capable of being transmitted by infected hosts to countries around the world, an important question for policy makers is whether or not these imported cases have the potential to generate sustained human-to-human transmission in new locations.

Here, we present data describing the times from symptom onset to hospitalisation for 47 patients from the current outbreak, obtained from publicly available line lists [15]. We fit an exponential distribution to these data, accounting for uncertainty due to the limited numbers of patients from whom data were available. Assuming that this distribution characterises the time spent by infected hosts generating new transmissions in the community, it is then possible to infer the probability that a case arriving in a new location is followed by an outbreak driven by sustained human-to-human transmission. We estimate this probability under the assumption that the transmissibility of the 2019-nCoV is similar to that of the SARS coronavirus, and then go on to consider the effect of shortening the mean time from symptom onset to hospitalisation. This provides an estimate of the risk that imported cases generate sustained outbreaks in new locations under different surveillance levels.

## 2. Methods

### 2.1. Time from Symptom Onset to Hospitalisation

**Figure 1:**
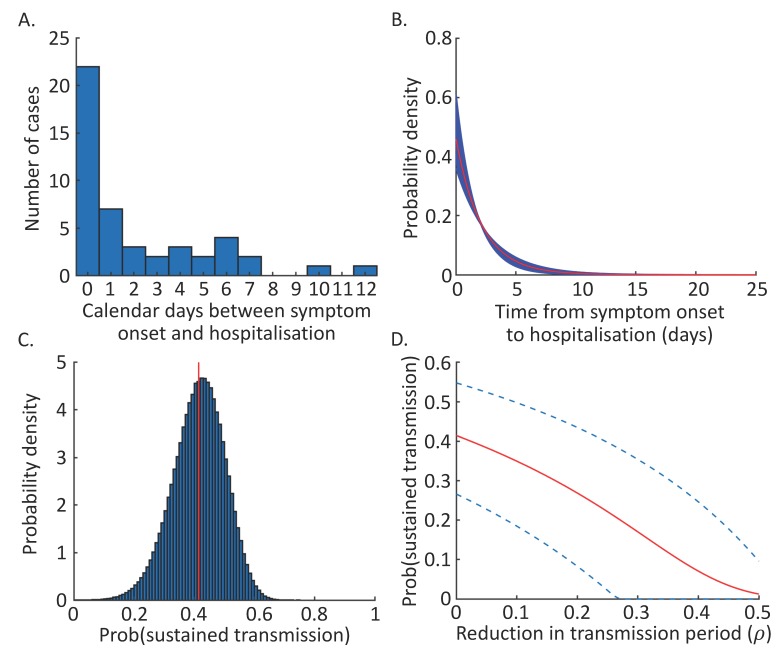
The probability of a self-sustaining outbreak driven by human-to-human transmission arising following the importation of one infected individual. (**A**) Data describing the number of days between symptom onset and hospitalisation for 47 patients in the ongoing outbreak [15]. (**B**) The estimated distribution of times between symptom onset and hospitalisation, obtained by fitting to the data shown in panel A. Blue lines show a range of equally possible distributions (see Methods; 50 distributions are shown here, selected at random from the *n* = 100,000 distributions considered), and the red line shows the average of the *n* = 100,000 distributions. (**C**) The probability of sustained transmission for each possible distribution of times from symptom onset to hospitalisation (Equation (1); blue histogram) and the probability of sustained transmission obtained by integrating over the possible distributions (Equation (2); red line). (**D**) The probability that a single imported case leads to sustained transmission in a new location, for different surveillance levels. The red line shows the mean estimates (obtained using Equation (2) but extended to account for intensified surveillance), and the blue dotted lines show the 5th and 95th percentile estimates (obtained when Equation (1) is applied, but extended to account for intensified surveillance).

The distribution of times from symptom onset to hospitalisation was estimated using patient data from the ongoing outbreak [15] (data are shown in Figure 1A). Since the precise times of symptom onset and hospitalisation on the dates concerned were unknown, we converted the times from symptom onset to hospitalisation to intervals describing possible time periods (see the Supplementary Data). For example, for a case showing symptoms on 9 January 2020, and then being hospitalised on 14 January 2020, the time between symptom onset and hospitalisation lies between four and six days (see e.g., Supplementary Material of [16] for a similar calculation). This is because the minimum possible period involves symptom onset at the end of 9 January and hospitalisation at the start of 14 January, whereas the maximum possible period involves symptom onset early on 9 January and hospitalisation late on 14 January.

We then fitted the rate parameter (γ) of an exponential distribution to these interval-censored data using Markov chain Monte Carlo (MCMC). A chain of length 10,000,000 in addition to a burn-in of 100,000 was used. The chain was then sampled with a thinning interval of 100 steps, giving rise *n* = 100,000 equally possible distributions for the times from symptom onset to hospitalisation, each characterised by a parameter estimate $\gamma_{i}$ (i=1,2,…,n). For further details of the MCMC algorithm used, see the Supplementary Text.

### 2.2. Estimating the Probability of Sustained Transmission

The distributions of times from symptom onset to hospitalisation were used to estimate the probability that an imported case will lead to sustained transmission, by assuming that infections occur according to a branching process (e.g., [17,18,19]). In this branching process, the effective reproduction number (accounting for control interventions, other than intensified surveillance which we model explicitly) of the 2019-nCoV when the virus arrives in a new location is denoted by R=β/γ, where the parameter β represents pathogen transmissibility [20]. We assumed that the transmissibility of the virus is similar to that of the SARS coronavirus, i.e., $\beta =R_{SARS}\gamma_{SARS}$, where $R_{SARS}=3$ [21] and the mean infection duration for SARS is 1/$\gamma_{SARS}=3.8$ days [22]. However, we adjusted the infectious period to account for the data describing the times between symptom onset and hospitalisation in the current outbreak. In doing this, we assumed that the time between an individual first displaying symptoms and being hospitalised was the period in which that individual was potentially transmitting the virus in the community.

The probability of a sustained transmission chain [19,20] starting from a single index case can be estimated for each of the equally possible distributions for the time from symptom onset to hospitalisation,

(1) $$\mathrm{Prob}(\mathrm{sustained}\;\mathrm{transmission}\;|\;\gamma_{i})=1-\frac{1}{\left(\beta /\gamma_{i}\right)}$$

In this expression, it is assumed that $\beta /\gamma_{i}>1$ (otherwise the probability of sustained transmission takes the value zero). If required, this can then be combined into a single estimate for the probability that an imported case leads to sustained transmission, p, given by

(2) $$p=\frac{1}{n}\overset{n}{\underset{i=1}{\sum }}\mathrm{Prob}(\mathrm{sustained}\;\mathrm{transmission}\;|\;\gamma_{i})$$

To include intensified surveillance in these estimates, we simply replaced the mean time from symptom onset to hospitalisation for each of the equally plausible distributions, $1/\gamma_{i}$, by the modified expression $\left(1-\rho \right)/\gamma_{i}$. In this approximation, the parameter ρ represents the proportional reduction in the mean infectious period due to intensified surveillance.

### 2.3. Multiple Imported Cases

The risk of sustained transmission given multiple imported cases was calculated by considering the possibility that none of those cases led to sustained transmission. Consequently,

(3) $$\mathrm{Prob}(\mathrm{sustained}\;\mathrm{transmission}\;|\;m\;\mathrm{imported}\;\mathrm{cases},\;\gamma_{i})=1-{\left(\frac{1}{\left(\beta \left(1-\rho \right)/\gamma_{i}\right)}\right)}^{m}$$

In this expression, it is assumed that $\beta \left(1-\rho \right)/\gamma_{i}>1$ (otherwise the probability of sustained transmission takes the value zero). Again, if required, this can be combined into a single estimate for the probability of sustained transmission starting from *m* imported cases, $p_{m}$, given by

(4) $$p_{m}=\frac{1}{n}\overset{n}{\underset{i=1}{\sum }}\mathrm{Prob}(\mathrm{sustained}\;\mathrm{transmission}\;|\;m\;\mathrm{imported}\;\mathrm{cases},\;\gamma_{i})$$

## 3. Results

As described in Methods, the distribution of times between symptom onset and hospitalisation was estimated using Markov chain Monte Carlo (Figure 1B and Figure S1) from the patient data in Figure 1A. This gave rise to a range of equally plausible distributions describing these time periods (blue lines in Figure 1B). The average of these distributions is shown by the red line in Figure 1B, however we used the full range of distributions in our calculations of the probability of sustained transmission occurring from each imported case.

Each of the range of plausible distributions corresponded to an estimate for the probability of a self-sustaining outbreak (Equation (1) and histogram in Figure 1C). A single estimate for the probability of sustained transmission can be obtained by summing over the range of distributions using Equation (2). The resulting probability of sustained transmission is 0.41 (red line in Figure 1C), with credible interval (CrI) [0.27, 0.55], where the CrI reflects the 5th and 95th percentile estimates.

We then considered the reduction in the probability that an imported case leads to sustained transmission if surveillance is more intense. Specifically, we assumed that intensified surveillance led to a reduction in the mean period from symptom onset to hospitalisation, governed by the parameter ρ (where ρ=0 corresponds to no intensification of surveillance, and ρ=1 corresponds to an implausible scenario in which infectious cases are hospitalised immediately). We found that, if surveillance is intensified so that the mean time from symptom onset to hospitalisation is halved, the probability that each imported case leads to sustained transmission is reduced to only 0.012 (CrI [0, 0.099]; Figure 1D).

**Figure 2:**
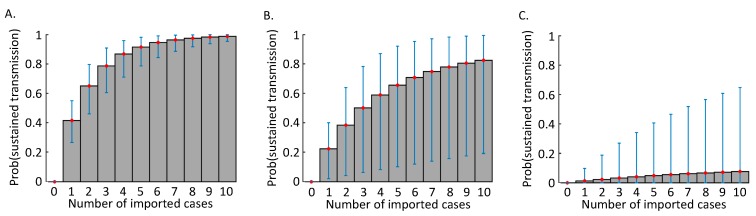
The probability of a self-sustaining outbreak driven by human-to-human transmission arising from multiple independent cases imported to a new location, under different surveillance levels. (**A**) No intensification of surveillance (ρ=0). (**B**) Medium level of surveillance intensification (ρ=0.25 ). (**C**) High level of surveillance intensification (ρ=0.5 ). The grey bars and red dots show the mean estimates (obtained using Equation (4)), and the error bars indicate the 5th and 95th percentile estimates obtained when Equation (3) is applied.

Finally, we considered the combined effect if multiple cases arrive in a new location. In that scenario, intense surveillance has the potential to reduce the risk of sustained transmission significantly compared to weak surveillance. For ρ = 0.5, the probability that any of 10 imported cases generate a substantial outbreak is only 0.12 (CrI [0, 0.65]; Figure 2C). This highlights the importance of rigorous surveillance, particularly in locations where infected hosts are most likely to travel.

## 4. Discussion

There are concerns that the ongoing outbreak driven by the 2019-nCoV could spread globally [3,5,23,24,25] with sustained transmission in countries around the world. Periods of high travel rates, such as the recent Chinese New Year holiday, present a significant challenge since they pose an elevated risk of importations of the virus to new locations [3,13]. In an effort to prevent a surge in travel, the Chinese government extended the national New Year holiday in January 2020.

Here, we have estimated the potential for cases arriving in new locations to lead to sustained transmission. According to the basic model that we have constructed, if surveillance levels are similar to those in China at the beginning of the current outbreak, and if this virus is similarly transmissible to the SARS coronavirus that spread globally in 2002–2003, then the probability that each imported infected case generates an outbreak due to sustained transmission is approximately 0.41 (CrI [0.27, 0.55]; Figure 1C). However, under a higher level of surveillance, the risk of sustained outbreaks is substantially lower (Figure 1D). This result is particularly striking when multiple independent cases travel to a new location, either simultaneously or in sequence (Figure 2). In that scenario, intensified surveillance is particularly important.

Our study involves the simplest possible model that permits the risk of sustained transmission to be estimated from the very limited data that have been collected in this outbreak until now. As additional information becomes available, it will be possible to estimate the risk of outbreaks in new locations with more precision. We made the assumption that symptom appearance coincides with the onset of infectiousness. One of the features of the SARS outbreak in 2002–2003 that allowed it to eventually be brought under control was the low proportion of onward transmissions occurring either prior to symptoms or from asymptomatic infectious hosts [26]. It might be hoped that infections due to the 2019-nCoV share this characteristic. Some reports have suggested that this may not be the case, although the extent of presymptomatic transmission is disputed [25,27]. We are working on an updated version of our analyses that includes the possibility of transmission from presymptomatic or mildly symptomatic hosts (based on the “SEAIR” compartmental model [19]).

Since our results were obtained using patient data from early in the ongoing outbreak, surveillance systems may not have been fully established when these data were collected, and patients may not have been primed to respond quickly to early symptoms. Our results might therefore be viewed as an upper bound on the risk posed by the 2019-nCoV. As the outbreak continues, it might be expected that the time from symptom onset to hospitalisation or isolation will decrease, leading to lower risks of sustained transmission, as has been observed for outbreaks of other diseases (e.g., the ongoing outbreak of Ebola virus disease in the Democratic Republic of the Congo). Initial indications suggest that such a decrease is occurring in China for this outbreak. In contrast, there may be some individuals that developed symptoms, but had not yet reported their infection by the time our analysis was conducted. “Right censoring” in this way favours lower reporting times, and so falsely reduces estimates of the time between symptom onset and hospitalisation [16,28].

Going forwards, it will be possible to include additional realism in the model. One example might be to consider spatial variation in host population densities and surveillance levels, leading to spatially inhomogeneous outbreak risks. Another possibility might be to account more explicitly for heterogeneities between different infectors, either by incorporating “superspreaders” [29] in the model or by differentiating between individuals that report disease at different rates. Such heterogeneity might be expected to reduce the risk of sustained transmission (for a preliminary analysis, in which individuals can either be “fast reporters” or “slow reporters”, see the Supplementary Text). It would also be possible to differentiate between mild and severe cases in the model. On the one hand, a mild case might be more likely to go unnoticed than a severe case, potentially leading to a higher outbreak risk. On the other hand, mild infections may generate fewer secondary cases than severe infections, thereby decreasing the outbreak risk. Complex interactions may therefore affect the risk of sustained transmission in an unpredictable fashion.

Despite the necessary simplifications made in this study, our analyses are sufficient to demonstrate the key principle that rigorous surveillance is important to minimise the risk of the 2019-nCoV generating large outbreaks in countries worldwide. We therefore support the ongoing work of the World Health Organization and policy makers from around the world, who are working with researchers and public health experts to manage this outbreak [2]. We also appreciate efforts to make data publicly available [15]. Careful analysis of the outbreak, as well as minimisation of transmission risk as much as possible, is of clear public health importance.

## Supplementary Materials

The following are available online at https://www.mdpi.com/2077-0383/9/2/498/s1.

## References

[1] Hui D.S., Azhar E.I., Madani T.A., Ntoumi F., Kock R., Dar O., Ippolito G., Mchugh T.D., Memish Z.A., Drosten C. (2020). The continuing 2019-nCoV epidemic threat of novel coronaviruses to global health—The latest 2019 novel coronavirus outbreak in Wuhan, China. Int. J. Infect. Dis..

[2] World Health Organization Novel Coronavirus (2019-nCoV); Situation Report 3; 2020. https://www.who.int/emergencies/diseases/novel-coronavirus-2019/situation-reports.

[3] Imai N., Dorigatti I., Cori A., Riley S., Ferguson N.M. (2020). Report 1: Estimating the Potential Total Number of Novel Coronavirus (2019-nCoV) Cases in Wuhan City, China. https://www.imperial.ac.uk/mrc-global-infectious-disease-analysis/news--wuhan-coronavirus/.

[4] Chan J.F.W., Yuan S., Kok K.H., To K.K.W., Chu H., Yang J., Xing F., Liu J., Chik-Yan Yip C., Wing-Shan Poon R. (2020). A familial cluster of pneumonia associated with the 2019 novel coronavirus indicating person-to-person transmission: A study of a family cluster. Lancet.

[5] Nishiura H., Jung S.M., Linton N.M., Kinoshita R., Yang Y., Hayashi K., Kobayashi T., Yuan B., Akhmetzhanov A.R. (2020). The extent of transmission of novel coronavirus in Wuhan, China, 2020. J. Clin. Med..

[6] Wu P., Hao X., Lau E.H.Y., Wong J.Y., Leung K.S.M., Wu J.T., Cowling B.J., Leung G.M. (2020). Real-time tentative assessment of the epidemiological characteristics of novel coronavirus infections in Wuhan, China, as at 22 January 2020. Eurosurveillance.

[7] Imai N., Cori A., Dorigatti I., Baguelin M., Donnelly C.A., Riley S., Ferguson N.M. (2020). Report 3: Transmissibility of 2019-nCoV. https://www.imperial.ac.uk/media/imperial-college/medicine/sph/ide/gida-fellowships/Imperial-2019-nCoV-transmissibility.pdf.

[8] Lipsitch M., Donnelly C.A., Fraser C., Blake I.M., Cori A., Dorigatti I., Ferguson N.M., Garske T., Mills H.L., Riley S. (2015). Potential Biases in estimating absolute and relative case-fatality risks during outbreaks. PLoS Negl. Trop. Dis..

[9] Ghani A.C., Donnelly C.A., Cox D.R., Griffin J.T., Fraser C., Lam T.H., Ho L.M., Chan W.S., Anderson R.M., Hedley A.J. (2005). Methods for estimating the case fatality ratio for a novel, emerging infectious disease. Am. J. Epidemiol..

[10] BBC (2020). China Coronavirus: UK Tracing up to 2000 Wuhan Visitors. https://www.bbc.co.uk/news/uk-51232163.

[11] Tatem A.J., Rogers D.J., Hay S.I. (2006). Global Transport Networks and Infectious Disease Spread. Adv. Parasitol..

[12] Thompson R.N., Thompson C., Pelerman O., Gupta S., Obolski U. (2019). Increased frequency of travel in the presence of cross-immunity may act to decrease the chance of a global pandemic. Philos. Trans. R. Soc. B.

[13] Cohen J., Normile D. (2020). New SARS-like virus in China triggers alarm. Science.

[14] Parry J. (2003). SARS virus identified, but the disease is still spreading. Br. Med. J..

[15] Kraemer M., Pigott D., Xu B., Hill S., Gutierrez B., Pybus O. (2020). Epidemiological Data from the nCoV-2019 Outbreak: Early Descriptions from Publicly Available Data. http://virological.org/t/epidemiological-data-from-the-ncov-2019-outbreak-early-descriptions-from-publicly-available-data/.

[16] Thompson R.N., Stockwin J.E., Gaalen R.D., Van Polonsky J.A., Kamvar Z.N., Demarsh P.A., Dahlqwist E., Li S., Miguel E., Jombart T. (2019). Improved inference of time-varying reproduction numbers during infectious disease outbreaks. Epidemics.

[17] Lloyd A.L., Zhang J., Root A.M. (2007). Stochasticity and heterogeneity in host-vector models. J. R. Soc. Interface.

[18] Allen L.J.S., Lahodny G.E. (2012). Extinction thresholds in deterministic and stochastic epidemic models. J. Biol. Dyn..

[19] Thompson R.N., Gilligan C.A., Cunniffe N.J. (2016). Detecting presymptomatic infection is necessary to forecast major epidemics in the earliest stages of infectious disease outbreaks. PLoS Comput. Biol..

[20] Thompson R.N., Jalava K., Obolski U. (2019). Sustained transmission of Ebola in new locations: More likely than previously thought. Lancet Infect. Dis..

[21] Wallinga J., Teunis P. (2004). Different epidemic curves for severe acute respiratory syndrome reveal similar impacts of control measures. Am. J. Epidemiol..

[22] Feng D., Jia N., Fang L.Q., Richardus J.H., Han X.N., Cao W.C., De Vlas S.J. (2009). Duration of symptom onset to hospital admission and admission to discharge or death in SARS in mainland China: A descriptive study. Trop. Med. Int. Heal..

[23] Bogoch I.I., Watts A., Thomas-Bachli A., Huber C., Kraemer M.U.G., Khan K. (2020). Pneumonia of unknown etiology in Wuhan, China: Potential for international spread via commercial air travel. J. Travel Med..

[24] Huang C., Wang Y., Li X., Ren L., Zhao J., Hu Y., Zhang L., Fan G., Xu J., Gu X. (2020). Clinical features of patients infected with 2019 novel coronavirus in Wuhan, China. Lancet.

[25] Thompson R.N. (2020). Pandemic potential of 2019-nCoV. Lancet Infect. Dis..

[26] Fraser C., Riley S., Anderson R.M., Ferguson N.M. (2004). Factors that make an infectious disease outbreak controllable. Proc. Natl. Acad. Sci. USA.

[27] Kupferschmidt K. Study Claiming New Coronavirus Can be Transmitted by People without Symptoms Was Flawed. https://www.sciencemag.org/news/2020/02/paper-non-symptomatic-patient-transmitting-coronavirus-wrong.

[28] Donnelly C.A., Ghani A.C., Leung G.M., Hedley A.J., Fraser C., Riley S., Abu-Raddad L.J., Ho L.M., Thach T.Q., Chau P. (2003). Epidemiological determinants of spread of causal agent of severe acute respiratory syndrome in Hong Kong. Lancet.

[29] Lloyd-Smith J.O., Schreiber S.J., Kopp P.E., Getz W.M. (2005). Superspreading and the effect of individual variation on disease emergence. Nature.

