# Supplementary material for: Novel Coronavirus Outbreak in Wuhan, China, 2020: Intense Surveillance Is Vital for Preventing Sustained Transmission in New Locations
Source: J Clin Med. 2020 Feb 11;9(2):498. doi: 10.3390/jcm9020498 (PMC7073840; doi:10.3390/jcm9020498)
Supplement: Supplementary file 1 [file jcm-09-00498-s001.zip › Supplementary Text.docx]

**Novel coronavirus outbreak in Wuhan, China, 2020: Intense surveillance is vital for preventing sustained transmission in new locations**

**Supplementary Text**

**AUTHOR**

R.N. Thompson^1,2,^*

*Correspondence to: [robin.thompson@chch.ox.ac.uk](mailto:robin.thompson@chch.ox.ac.uk)

**AFFILIATIONS**

^1^Mathematical Institute, University of Oxford, Woodstock Road, Oxford OX2 6GG, UK

^2^Christ Church, University of Oxford, St Aldates, Oxford OX1 1DP, UK

**Estimating the time from symptom onset to hospitalisation**

In the main text, we described estimation of the distribution of times from symptom onset to hospitalisation. This involved using Markov chain Monte Carlo to estimate the rate parameter, $\gamma$, of an exponential distribution from interval-censored data. In each step of the chain, we either (with probability 0.5 each) propose a new value of the parameter $\gamma$ (sampled uniformly from the range [0.09, 2.33], which corresponds to periods between one fifth and five times the mean observed period in Fig 1A) or we propose new times between symptom onset and hospitalisation for all of the 47 patients (sampled uniformly from the possible periods in the Supplementary Data). The proposed value of $\gamma$ or times between symptom onset and hospitalisation are then either accepted or rejected, based on the exponential likelihoods corresponding to the current and proposed states of the chain.

In Fig S1, we show the trace plot for this chain, after removal of the burn-in period and after thinning as described in the main text.

Figure S1. Trace plot for estimation of the rate of symptom onset to hospitalisation using Markov chain Monte Carlo. This trace plot corresponds to the fitted distributions shown in Fig 1B of the main text. Dotted red lines show the boundaries of the uniform prior used for $\gamma$.

**Extending our approach to more complex models**

Here we show how our analysis can be extended for models that are more complex than the basic exponential model considered in the main text. In Fig 1A of the main text, it can be seen that a large number of individuals report to hospital on the same day that they develop symptoms. For that reason, here we consider an extended model in which infected individuals are either “fast reporters” or “slow reporters”.

Specifically, we assume that a proportion $\alpha$ of infected individuals are likely to have a short period between symptom onset and hospitalisation, with mean $1/\gamma^{(1)}$ days. The remaining $1-\alpha$ of individuals are likely to have a longer reporting period, with mean $1/\gamma^{(2)}$ days. These two groups could be viewed as patients that are inclined to report disease (fast reporters), and those that are not inclined to report disease (slow reporters), respectively. As a result, $\gamma^{(1)}$ > $\gamma^{(2)}$.

We then estimated the values of $\alpha$, $\gamma^{(1)}$ and $\gamma^{(2)}$ using Markov chain Monte Carlo from the same interval-censored data that were used for our simpler analysis in the main text (Supplementary Data). In each iteration of the chain, we chose at random (each with one-third probability) whether to: i) sample the periods between symptom onset and hospitalisation for every individual (uniformly at random from the possible periods in the Supplementary Data); ii) sample new possible values of $\gamma^{(1)}$ and $\gamma^{(2)}$ (both uniformly in the range [0.04, 46.5]); iii) sample a new possible value of $\alpha$ (uniformly in the range [0,1]). In case two, we randomised whether the value of $\gamma^{(1)}$ or $\gamma^{(2)}$ was sampled first, and then restricted the second sampled value to ensure that $\gamma^{(1)}$ > $\gamma^{(2)}$. We used the same burn-in period and thinning procedure as for the original model.

We then conducted the analogous analysis to the one shown in Fig 1 of the main text. To calculate the probability of sustained transmission starting from a single infected individual, who could either be a fast reporter or a slow reporter, we used the formula

$$Prob(sustained transmission)=Prob(sustained transmission|index case is fast reporter)\times Prob(index case is fast reporter)+Prob(sustained transmission|index case is slow reporter)\times Prob(index case is slow reporter).$$

Assuming the index case is chosen randomly from all individuals, then $Prob(index case is fast reporter)$ takes the value $\alpha$, and $Prob(index case is slow reporter)$ takes the value $1-\alpha$. To calculate $Prob(sustained transmission|index case is fast reporter)$ and $Prob(sustained transmission|index case is slow reporter)$, we denoted by $q_{i,j}$ the probability of not seeing a chain of sustained transmission starting from *i* fast reporters and *j* slow reporters. Then, starting from either one fast reporter or one slow reporter, and conditioning on whether the first event is an infection event or a recovery event, gives

$$q_{1,0}=\frac{\alpha\beta}{\beta+\gamma^{(1)}}q_{2,0}+\frac{(1-\alpha)\beta}{\beta+\gamma^{(1)}}q_{1,1}+{\frac{\gamma^{(1)}}{\beta+\gamma^{(1)}}q}_{0,0},$$

$$q_{0,1}=\frac{\alpha\beta}{\beta+\gamma^{(2)}}q_{1,1}+\frac{(1-\alpha)\beta}{\beta+\gamma^{(2)}}q_{0,2}+{\frac{\gamma^{(2)}}{\beta+\gamma^{(2)}}q}_{0,0.}$$

Assuming that infection lineages deriving from different infected hosts are independent (so that, e.g., $q_{1,1}=q_{1,0}q_{0,1}$), and noting that $q_{0,0}=1$, gives

$$q_{1,0}=\frac{\alpha\beta}{\beta+\gamma^{(1)}}{q_{1,0}}^{2}+\frac{(1-\alpha)\beta}{\beta+\gamma^{(1)}}q_{1,0}q_{0,1}+\frac{\gamma^{(1)}}{\beta+\gamma^{(1)}},$$

$$q_{0,1}=\frac{\alpha\beta}{\beta+\gamma^{(2)}}q_{1,0}q_{0,1}+\frac{(1-\alpha)\beta}{\beta+\gamma^{(2)}}{q_{0,1}}^{2}+\frac{\gamma^{(2)}}{\beta+\gamma^{(2)}}.$$

These two simultaneous equations can be solved for $q_{1,0}$ and $q_{0,1}$, and then

$$Prob(sustained transmission|index case is fast reporter)=1-q_{1,0},$$

$$Prob(sustained transmission|index case is slow reporter)=1-q_{0,1}.$$

In these expressions, we take the solutions for $q_{1,0}$ and $q_{0,1}$ that lie in the range (0,1). If such values do not exist, then solutions for $q_{1,0}$ and $q_{0,1}$ equal to one are used.

In Fig S2A, we show the distributions of times between symptom onset and hospitalisation for slow reporters (black line), fast reporters (blue line) and all individuals (red line). These distributions are analogous to the red line in Fig 1B of the main text for each of these groups. In Fig S2B, we show the mean probability of sustained transmission starting from a single index case. This is analogous to the red line in Fig 1D of the main text. By comparing Fig S2B and Fig 1D, it can be seen that, in the absence of intensified surveillance, heterogeneity in the numbers of secondary cases arising from each infector acts to reduce the probability of a sustained outbreak occurring.


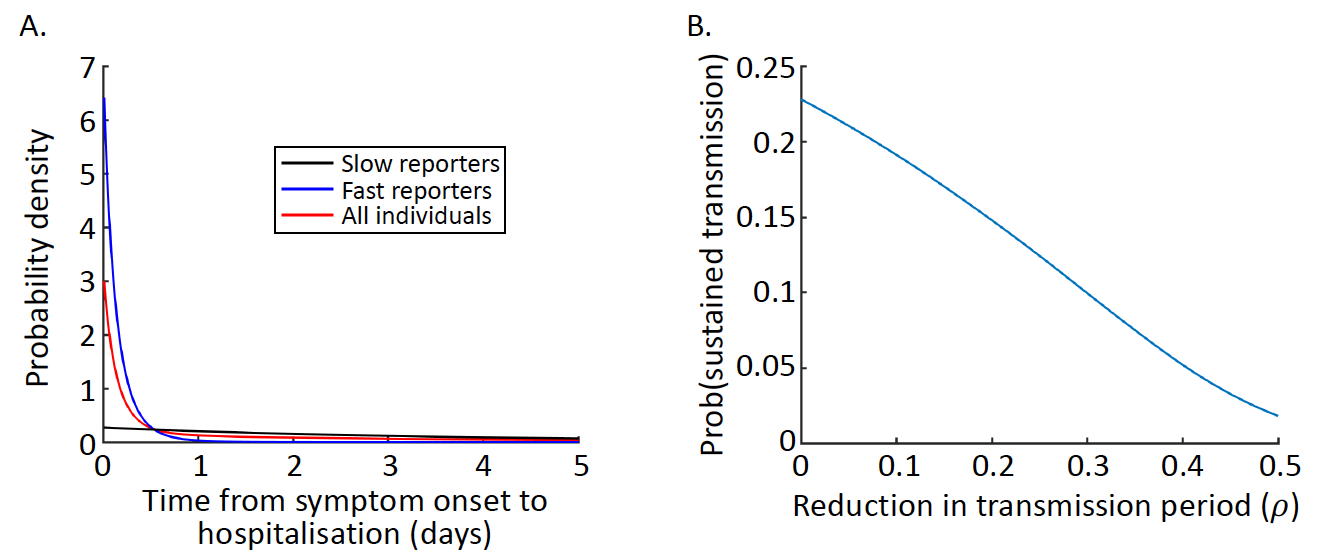


Figure S2. The probability of a self-sustaining outbreak driven by human-to-human transmission arising following the introduction of one infected individual, according to a model consisting of “fast reporters” and “slow reporters”. A. The estimated distribution of times between symptom onset and hospitalisation, obtained by fitting to the data shown in Fig 1A of the main text. Model fits are shown for slow reporters (black), fast reporters (blue) and all reporters (red). The distributions shown are averaged over the *n* = 100,000 distributions obtained from the MCMC fitting, but the full set of distributions are used to estimate the probability of sustained transmission. The estimated mean values of the time from symptom onset to reporting for fast reporters and slow reporters are 1/$\gamma^{(1)}$ = 0.19 days and 1/$\gamma^{(2)}$ = 3.61 days, respectively. The x-axis is curtailed at 5 days so that the distributions can be seen more easily. B. The probability that a single imported case leads to sustained transmission in a new location, for different surveillance levels. The calculation performed is analogous to equation (2) of the main text, extended to include intensified surveillance and accounting for the possibility that the initial case could be either a slow reporter or a fast reporter.
